# Anticancer Molecular Mechanisms of Curcuminoids: An Updated Review of Clinical Trials

**DOI:** 10.1002/fsn3.71452

**Published:** 2026-02-03

**Authors:** Ushna Momal, Muhammad Shahbaz, Asfa Perween, Muhammad Hammad ul Hassan, Hammad Naeem, Zubda Shahid, Muzzamal Hussain, Muhammad Imran, Suliman A. Alsagaby, Waleed Al Abdulmonem, Mohamed A. Abdelgawad, Mohammed M. Ghoneim, Tadesse FentaYehuala, Samy Selim, Ehab M. Mostafa

**Affiliations:** ^1^ Department of Food Science and Technology Muhammad Nawaz Shareef Univerity of Agriculture Multan Pakistan; ^2^ Department of Human Nutrition and Dietetics Muhammad Nawaz Shareef University of Agriculture Multan Pakistan; ^3^ Department of Biochemistry Avicenna Medical College Lahore Pakistan; ^4^ Department of Food Science Government College University Faisalabad Faisalabad Pakistan; ^5^ Department of Food Science and Technology University of Narowal Narowal Pakistan; ^6^ Department of Medical Laboratory Sciences, College of Applied Medical Sciences Majmaah University AL‐Majmaah Saudi Arabia; ^7^ Department of Pathology, College of Medicine Qassim University Buraidah Kingdom of Saudi Arabia; ^8^ Department of Pharmaceutical Chemistry, College of Pharmacy Jouf University Sakaka Aljouf Saudi Arabia; ^9^ Department of Pharmacy Practice, College of Pharmacy AlMaarefa University Riyadh Saudi Arabia; ^10^ Faculty of Chemical and Food Engineering, Bahir Dar Institute of Technology Bahir Dar University Bahir Dar City Ethiopia; ^11^ Department of Clinical Laboratory Sciences, College of Applied Medical Sciences Jouf University Sakaka Saudi Arabia; ^12^ Department of Pharmacognosy, College of Pharmacy Jouf University Sakaka Saudi Arabia; ^13^ Pharmacognosy and Medicinal Plants Department, Faculty of Pharmacy (Boys) Al‐Azhar University Cairo Egypt

**Keywords:** apoptosis, chemotherapy, curcumin, extracellular matrix, inflammation, metastasis, nanoparticles, polyphenolic substances, signaling pathways

## Abstract

Curcuminoids are bioactive polyphenols, mainly extracted from *Curcuma longa* (turmeric), and have received much attention due to their pleiotropic anticancer properties. Recent evidence suggests that curcumin and analogs prevent the activation of a variety of signaling pathways, including the MAPK, PI3K/Akt, and NF‐kB pathways, resulting in the induction of apoptosis, prevention of proliferation, and suppression. In addition to these processes, curcumin has been shown to enhance the efficacy of the conventional anti‐cancer modalities such as radiation and chemotherapy. By suppressing the expression of matrix metalloproteinases (MMPs), which are enzymes that break down the extracellular matrix and promote cancer cell invasion and metastasis, curcumin also demonstrates anti‐metastatic qualities. Inflammation and the advancement of cancer are linked to the NF‐κB signaling pathways, which are also suppressed by curcuminoids. Along with these processes, curcumin has demonstrated the ability to improve the effectiveness of traditional cancer treatments, including radiation and chemotherapy. It increases the sensitivity of cancer cells to various therapies, which enhances the therapeutic results. However, curcumin's low bioavailability limits its therapeutic use. Its anticancer potency may be increased, and this restriction may be overcome due to recent developments in drug delivery technologies, such as curcumin‐loaded nanoparticles. Since they can target several different molecular pathways and improve the effectiveness of current treatments, curcuminoids are a promising family of chemicals for the prevention and treatment of cancer.

## Graphical abstract

Curcuminoids remain one of the most studied dietary polyphenols as a cancer prevention and treatment agent. Inflammation and tumor progression are related to the NF‐κB signaling pathway, which is also suppressed by curcuminoids. Curcuminoids prevent the activation of a variety of signaling pathways, including the MAPK, PI3K/Akt, and NF‐kB pathways, resulting in the induction of apoptosis, prevention of proliferation, and suppression.

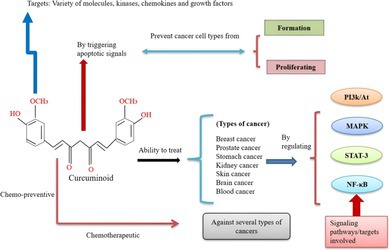

## 1 Introduction

**FIGURE 1:**
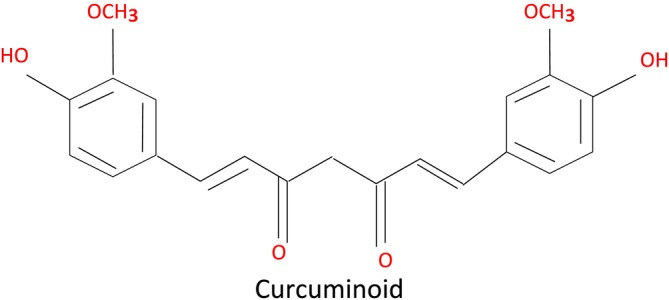
Chemical structure of curcuminoids.

Cancer is also one of the most common causes of global death, and the constraints of traditional therapeutic agents, such as toxicity, resistance, and recurrence, have increased the need for natural‐derived products with multiple targets. These include the curcuminoids, especially curcumin, demethoxycurcumin, and bis‐demethoxycurcumin, which have been widely studied in relation to their chemopreventive and therapeutic effects. The most examined curcuminoid and the most popular polyphenolic chemical, curcumin, is well known. Curcumin essentially has two feruloyl groups joined to a methylene bridge via α, β‐unsaturated carbonyl function. The chemical structure of curcuminoids is shown in Figure 1.

Curcuminoids are mainly derived from turmeric with an average weight to curcumin content of between 2% and 5% (w/w) (Fuloria et al. 2022). Some significant sources are as follows: Specific Curcuma types and varieties contain differing levels of curcuminoids: *Curcuma aromatica* and *Curcuma zedoaria* . The health benefits of curcumin extracts are also beneficial from its standardization, which provides it in concentrated forms (Farghadani and Naidu 2021). Curcumin, the most effective curcuminoid in this spice, has several health benefits due to its inflammation‐reducing and antioxidant properties. Demethoxycurcumin and curcumin are closely related but have properties that differ quite a lot, as well as have different biological effects, and in some cases are even more active. Bisdemethoxycurcumin: While not as common as other forms of this compound, curcumin exhibits reliably potent anti‐inflammatory and anti‐cancer qualities, which add significantly to turmeric's efficacy. These beneficial ingredients work synergistically, making turmeric a precious natural ingredient for health improvement (Bertazza et al. 2019). azOxidative stress refers to a physiological state caused by an imbalance between the production of reactive oxygen species (ROS) and the body's antioxidant defense mechanisms, leading to cellular and molecular damage. This imbalance contributes to chronic inflammation, DNA damage, and disease progression (Chen et al. 2022). Studies have shown that excessive ROS generation can activate pro‐inflammatory signaling cascades such as the NF‐κB and STAT3 pathways, both of which are central to the development of diabetes and cancer. Natural compounds like curcumin and resveratrol can reduce oxidative stress by suppressing these pathways and restoring redox balance (Abdollahi et al. 2021).

**FIGURE 2:**
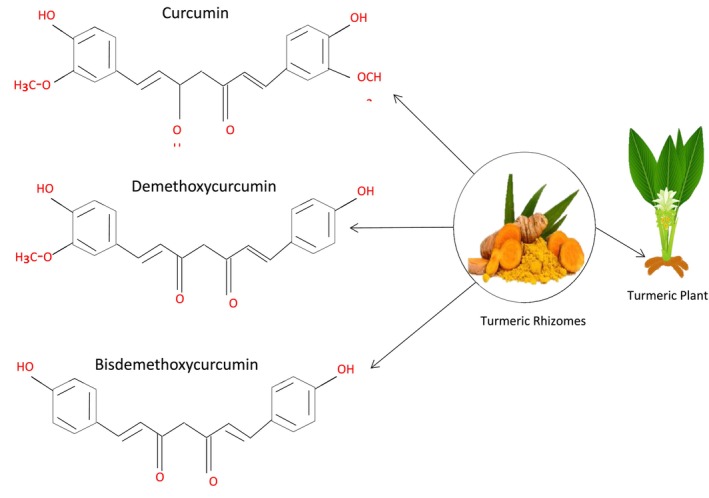
Chemical structures of the main types of curcuminoids in turmeric.

To provide a quantitative overview, recent meta‐analyses have statistically assessed curcumin's effectiveness and safety in treating various tumor types. Zhou et al. (2023) reported a pooled standardized mean difference (SMD) of −0.64 (95% CI: −0.82 to −0.47; *p* < 0.001) for reducing inflammatory biomarkers (TNF‐α, IL‐6, CRP) across 14 clinical studies, indicating moderate to vigorous anti‐inflammatory activity. Gupta et al. (2022) analyzed 11 randomized controlled trials in solid tumors. They found a pooled hazard ratio (HR) for overall survival of 0.82 (95% CI: 0.69–0.97), suggesting a modest yet statistically significant survival benefit with curcumin supplementation. Conversely, Hewlings et al. (2021) observed high heterogeneity (I^2^ = 71%) among studies caused by differences in formulation, dosage, and cancer subtype, underscoring the need for standardized trial designs. Overall, these quantitative results confirm that curcuminoids have measurable biological effects, although clinical benefits may vary depending on the context and formulation. An overview of the chemical structures of the main types of curcuminoids found in turmeric is shown in Figure 2.

Studies indicated the efficacy of an extraordinary class of compounds referred to mainly as curcuminoids. Because of their many mechanisms of action and diverse origins, they represent promising therapeutic agents in modern medicine. However, their therapeutic efficacy is not universal and can vary significantly depending on cancer type, tumor microenvironment, genetic alterations, and formulation bioavailability. Evidence from both preclinical and clinical studies indicates that curcuminoid responsiveness is context‐dependent rather than consistent across malignancies (Yue et al. 2023). Because of this, more research is required to fully comprehend the mechanisms for the beneficial effects of curcuminoids, enhance bioavailability, and determine potential therapeutic uses. While preclinical and clinical findings collectively support the anticancer potential of curcuminoids, the overall strength of evidence remains heterogeneous across studies and cancer types. A critical examination of published trials reveals that many exhibit small sample sizes, lack randomization, or use non‐standardized curcumin formulations, limiting comparability and reproducibility (Jabczyk et al. 2022). Some investigations report significant reductions in inflammatory biomarkers and tumor growth, whereas others fail to demonstrate measurable clinical benefit, particularly in advanced‐stage disease (Rahmani et al. 2023). Despite compelling preclinical data, clinical translation has been inconsistent, mainly due to poor systemic bioavailability and limited formulation stability. Addressing this gap, recent years have seen the emergence of nanoparticle, liposomal, and phospholipid‐complex formulations designed to enhance absorption and tissue distribution (Siddiqi et al. 2018).

Although initial selection criteria excluded studies mainly focused on formulation development and pharmaceutical design, nanoparticle and liposomal formulations were included in this review only when they directly evaluated biological or clinical outcomes, such as improved bioavailability, efficacy, or safety. This approach ensures consistency between the scope and the evidence discussed, keeping the focus on the molecular and therapeutic significance of curcuminoids rather than on formulation. To position curcuminoids within the broader spectrum of phytochemical anticancer research, this review also integrates insights from structurally distinct but mechanistically convergent compounds: ellagic acid—demonstrated to suppress breast cancer proliferation via modulation of estrogen receptor and oxidative pathways—and naringenin, which exerts colorectal anticancer effects through MAPK inhibition and modulation of cell‐cycle regulators. Including these studies broadens the molecular narrative and underscores the shared signaling frameworks exploited by plant‐derived polyphenols. The objectives of this updated review are therefore to appraise recent mechanistic evidence concerning curcuminoids critically, synthesize findings from available clinical trials in human populations, identify translational limitations such as bioavailability and heterogeneity of outcomes, and propose future research directions for improved clinical applicability (Li et al. 2023).

## 2 Antioxidant Potential of Curcuminoids

Antioxidants are most important in combating free radicals, which are chemicals that are highly reactive and cause harm to cells and could be involved in causing diseases, including cancer, cardiovascular diseases, and neurological disorders (Jakubczyk et al. 2020). The molecular formulation of curcuminoids profiles characterized them as antioxidants. Curcumin can easily donate hydrogen atoms, eliminate free radicals, and reduce oxidative stress due to the diketone and phenolic hydroxyl group. Due to many hydroxyl groups, curcumin is an antioxidant that has exceptionally high electron‐donating ability (Giordano and Tommonaro 2019). Curcuminoids have antioxidant effects through several metabolic processes and signaling systems. To identify these pathways, protective effects against further oxidative stress must be understood. Oxidative stress is protected through the main Nrf2 pathway that is found in cells. Under normal circumstances, Nrf2 exists in the cytoplasm bound to KEAP1 and thus targeted for degradation. However, curcumin helps Nrf2 dissociate from its inhibitor when the body is challenged with oxidative stress, where the protein accumulates in the nucleus. Once it gets into the nucleus, Nrf2 binds to antioxidant response elements (ARE) in the promoter region of several cytoprotective genes, thus inducing the activity of antioxidant enzymes, including superoxide dismutase and glutathione peroxidase (Smith et al. 2016).

Furthermore, it has been shown that curcumin can affect the Mitogen‐Activated Protein Kinase (MAPK) signal transduction, which is involved in signal transduction in cellular stress response pathways. Curcumin can modulate this pathway in a way that results in decreased oxidative stress and increased antioxidant proteins. These findings have shown that curcumin elevated antioxidant protection due to the activation of the p38 MAPK signaling pathway, which can potentially protect different types of cells (Sathyabhama et al. 2022). Curcumin is involved in direct free radical scavenging activity of several free radicals, including superoxide anions, hydroxyl radicals, and peroxyl radicals. Both the phenolic and diketone groups of curcumin, containing an electron‐rich nature, retain these reactive species. Many studies have pointed out curcumin's potential to reduce oxidative stress markers significantly in various biological models. Scientists have indicated that curcumin can considerably decrease oxidative stress indicators in a variety of biological systems (Giordano and Tommonaro 2019). A critical study that examined the way curcumin influenced oxidative stress in human endothelial cells was published. The researchers tried to assess the mechanisms that prevent oxidation enabled by curcumin. Furthermore, before the treatment with H_2_O₂, human umbilical vein endothelial cells (HUVECs) were treated with curcumin at the concentrations of 5, 10, and 20 μM. The investigation determined MDA, SOD, Nrf2, and HO‐1 levels as markers of oxidative stress. A significant decrease in the level of MDA in treated animals was observed, which indicated that curcumin treatment delays the process of lipid peroxidation. In the treated cells, a significant increase in the activity of the antioxidant enzyme SOD was observed. This improvement demonstrated that curcumin enhances the ability of cellular antioxidant defenses in addition to acting as a free radical scavenger. They are crucial for preventing oxidation and maintaining overall health since radicals quickly enter the bloodstream and harm the organism. This method focuses on curcuminoids for the treatment of diseases related to oxidative stress. It can be suggested that additional studies focusing on their scavenging properties can provide a better understanding of their role in maintaining health and preventing diseases (Shahcheraghi et al. 2021).

## 3 Pharmacokinetic Study of Curcuminoids

Curcuminoids are bioactive chemicals that are mainly recognized for anti‐inflammatory, antioxidant, and anticancer properties and are the focal point in medical science. Their pharmacokinetic properties, however, make them suitable for therapeutic applications. Curcumin has a short biological half‐life and is poorly soluble in water, which allows understanding the low bioavailability of curcumin after oral administration. As found out in a similar study, curcumin is widely metabolized in the liver and the intestinal wall when absorbed orally; the concentration of the drug in the plasma is very low (Bahramsoltani et al. 2019).

After intake, curcumin is broadly distributed throughout the body, with the kidneys, liver, and gastrointestinal tract containing the most significant quantities. However, its distribution is limited due to its high metabolism and clearance. Since curcumin is fat‐soluble, it can easily diffuse through cell membranes, and this is one of the reasons why the body gets rid of it so fast (Sohn et al. 2021). Curcumin is highly metabolized through conjugation pathways and through reduction pathways. The primary metabolites are dihydrocurcumin, tetrahydrocurcumin, and the glucuronide and sulfate derivatives of both dihydrocurcumin and tetrahydrocurcumin. Despite this fact, these metabolites are not as active as the parent chemical; they can possess some biological activity. As can be seen, curcumin is metabolized by cytochrome P450 enzymes and therefore could affect medication interactions. An average of 43.2% of curcumin and 55% of its metabolites were eliminated through feces, while only a moderate percentage of 16.1% and 8% of the total was found in urine. Due to its rapid elimination from the systemic circulation, normal doses or the development of sustained‐release preparations are needed to obtain therapeutic concentrations (Kotha and Luthria 2019).

Pharmacokinetic properties, safety, and therapeutic potential of curcumin in breast cancer patients have been evaluated in a study. In the study, curcumin was used in the form of nanoparticles so that the bioavailability of the nutrient would improve. The intake of 300 mg of curcumin nanoparticles was administered each day for 6 months. When the data was compared to 60% of the participants, there was an observed shrinkage of tumor size along with an elevated level of plasma curcumin (Sharifi‐Rad et al. 2021).

In clinical oncology, curcuminoids also pose significant pharmacokinetic and drug–drug interaction challenges that require caution. Curcumin is known to affect cytochrome P450 isoenzymes, specifically CYP3A4, CYP1A2, and CYP2C9, and P‐glycoprotein transporters, which can change the metabolism of chemotherapeutics and supportive drugs like paclitaxel, doxorubicin, and tamoxifen. Combining these may increase systemic drug levels, potentially raise toxicity, or change therapeutic effectiveness (Gupta et al. 2021). Additionally, curcumin's inhibitory effects on UDP‐glucuronosyltransferases can disrupt glucuronidation pathways vital for drug clearance, especially in cases involving multiple medications. Therefore, while improved formulations enhance bioavailability, their use in clinical practice requires careful pharmacovigilance, therapeutic drug monitoring, and assessment of possible adverse pharmacokinetic interactions (Pires et al. 2023).

A clinical trial investigated the pharmacokinetics of curcumin carried out among patients diagnosed with colorectal cancer. In the trial, a liposomal curcumin was used; the daily dose was 200 mg. It also manifested higher plasma concentration and improved bioavailability of curcumin by the same research. Patients also reported a reduced size of their tumor and inflammation because of taking the extract (de Porras et al. 2021; De Porras et al. 2021). A clinical experiment was conducted to investigate the pharmacokinetics of dimethoxycurcuminoids in advanced pancreatic cancer patients. The objectives of the study were to establish the therapeutic effectiveness of dimethoxycurcuminoids and ADME profiles. Two hundred milligrams of dimethoxycurcuminoids were given to patients daily for 12 weeks. The substances were distributed throughout the body, but they were most concentrated in the tissues of tumors and in the liver. While 40% had a partial response, their tumor sizes were significantly reduced. Out of the patients, 30% experienced stable disease with no change or deterioration. The study presented here shows that dimethoxycurcuminoids possess favorable pharmacokinetic properties and promising therapeutic potential in the treatment of metastatic pancreatic cancer. Some dimethoxycurcuminoids can be considered for further clinical investigation because of enhanced bioavailability and stability (Smith et al. 2022).

A major challenge in interpreting curcumin's clinical performance is the heterogeneity of trial endpoints, patient cohorts, and cancer subtypes investigated. Recent randomized and non‐randomized studies demonstrate variable outcomes ranging from biochemical response (e.g., PSA or cytokine reduction) to clinical efficacy endpoints like progression‐free survival (PFS) or overall survival (OS). Panahi et al. (2022) reported that curcumin supplementation in breast cancer patients led to a significant decrease in TNF‐α and IL‐6 levels, but no improvement in overall survival. In contrast, Kumar et al. (2021) observed enhanced PFS when curcumin was co‐administered with docetaxel in prostate cancer. Likewise, Rahmani et al. (2023) found clinical benefit in inflammation and VEGF reduction without radiographic tumor regression. These inconsistencies may stem from formulation‐dependent bioavailability, differing endpoints (biochemical vs. clinical), and variations in baseline disease stages.

## 4 Anticancer Prospects of Curcuminoids

**FIGURE 3:**
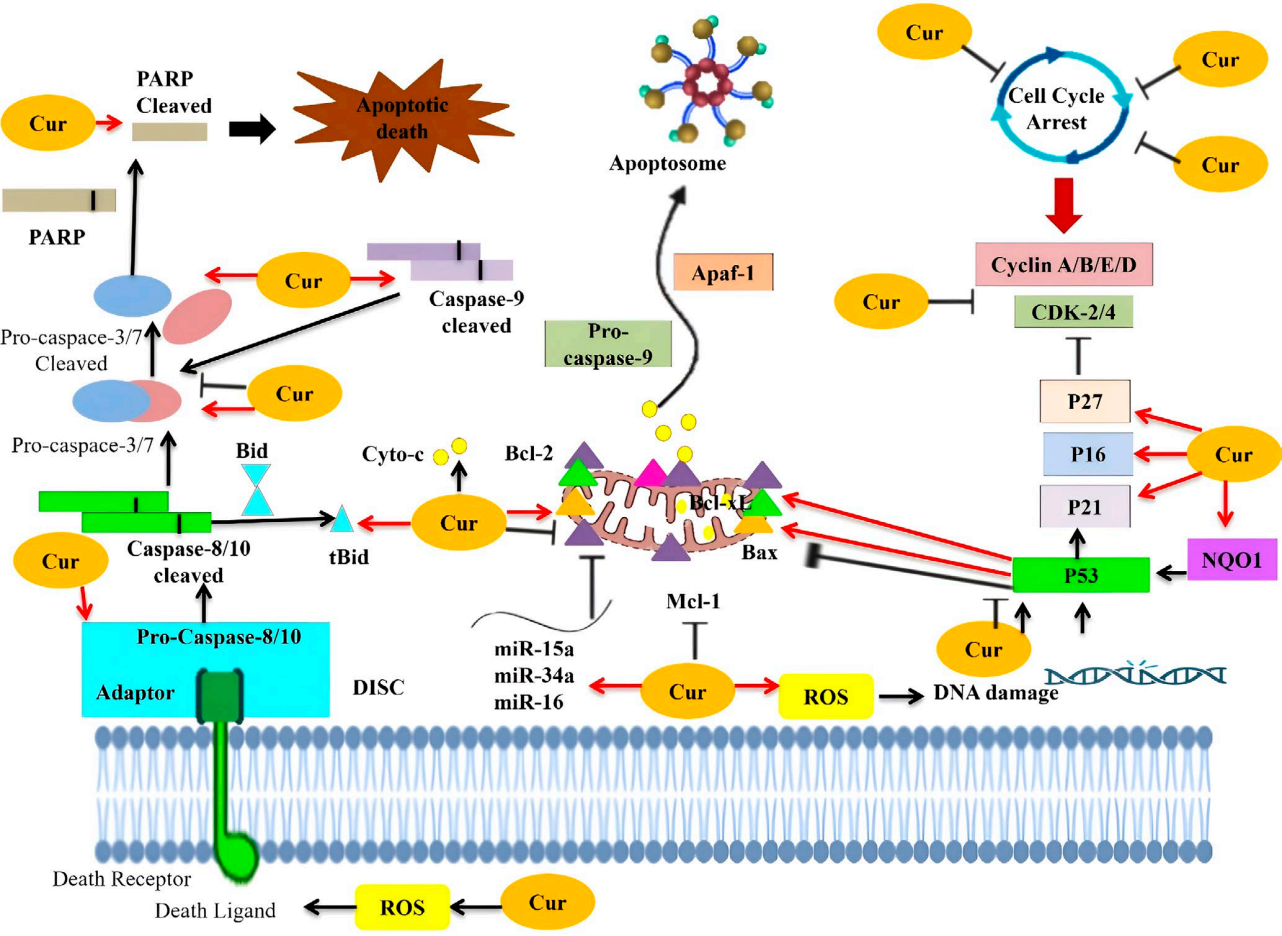
The modulatory effect of curcuminoids on p53 cell cycle and apoptosis pathways.

Curcuminoids, mainly curcumin, derived from turmeric, possess significant potential in cancer treatment because of their pharmacological activities. Anticancer potential of curcumin is through the ability to alter signal transduction, promote apoptosis, and inhibit angiogenesis and metastasis. This review focuses on the latest in vitro and in vivo investigations that describe the molecular actions through which curcuminoids help to prevent or treat cancer surgical effects. Curcumin has demonstrated anticancer properties through its capacity to modulate cellular signaling pathways, induce apoptosis, inhibit angiogenesis, and suppress metastasis. This review explores recent in vitro and in vivo studies that elucidate the molecular mechanisms of curcuminoids in combating cancer. Curcuminoids influence various oncogenic signal pathways to take charge of cell survival, growth, and metastasis. The key targets are the MAPK, PI3K/Akt, NF‐0, JAK/STAT, and Wnt/‐catenin pathways. The regulation of these cascades restores apoptotic balance, inhibits angiogenesis, and inhibits epithelial‐to‐mesenchymal transition (EMT) by curcuminoids. As an example, researchers found out that curcumin suppressed the signaling pathway of PI3K/AKT/mTOR in prostate cancer cells, which resulted in G1 cell cycle arrest (Imran et al. 2018). In another study, the researchers also found that curcumin has shown growth‐inhibitory effects on breast cancer cells, arresting the MAPK and NF‐κB pathways and restricting cell growth and proliferation. This compound is also known to increase the cytotoxicity of the standard treatment drugs and increase the rate at which cancer cells are killed. The researchers conducted an in vitro experiment, which suggested that curcumin suppressed MDR transporters and decreased the efflux of the drug; therefore, it increases the sensitivity of breast cancer cells to paclitaxel. The results demonstrated that this combination treatment enhanced the cytotoxic effects of paclitaxel and indicated that curcumin might be a helpful adjuvant in cancer therapy (Rodrigues et al. 2019). The modulatory effects of curcuminoids on the p53 cell cycle and apoptosis pathways are illustrated in Figure 3.

Curcuminoids modulate shared signaling cascades such as NF‐κB, MAPK, and PI3K/Akt; their mechanistic focus varies across different cancer types. In breast cancer, curcumin mainly targets PI3K/Akt/mTOR and estrogen receptor–related pathways, reducing cell proliferation and overcoming hormone resistance (Huang et al. 2023). In colorectal cancer, the central suppression occurs in Wnt/β‐catenin and NF‐κB signaling, leading to decreased β‐catenin nuclear translocation and increased apoptosis (Yadav et al. 2020). In prostate cancer, curcuminoids decrease androgen receptor signaling and inhibit NF‐κB–mediated inflammation and cell survival (Abd. Wahab et al. 2020). For glioblastoma, modulation of MAPK and Notch signaling promotes apoptosis and boosts chemosensitivity (He et al. 2019). In skin cancers, including melanoma, curcuminoids block the BRAF/MEK/ERK cascade, causing cell‐cycle arrest through p21/p27 activation and CDK4 suppression. These different mechanistic signatures show that the anticancer effects of curcuminoids are highly dependent on context, influenced by genetic mutations, tumor microenvironment, and dominant receptor signaling in each cancer type (Choudhury et al. 2024a, 2024b).

For instance, a study showed that treatment with curcumin caused a significant decrease in tumor size and its weight in a mouse model of colon cancer. Tumor growth was suppressed by the modulation of the NF‐*k*B signaling pathway and by promotion of apoptosis through activation of caspase 3 (Wang et al. 2019). Researchers have shown that curcumin exhibited a similar blocking effect on mouse models of breast cancer xenografts. The study pointed out the anticancer effects of curcumin‐mediated suppression of cyclin D1, a protein whose overexpression leads to cell cycle progression (Xiang et al. 2020).

Moreover, it has been found that curcumin succeeds in overcoming chemoresistance in animals and has direct anticancer activity. Curcumin was shown to enhance the ability of cisplatin to affect ovarian cancer cells in a mouse model. The curcumin co‐treatment had a lesser tumor volume as compared with cisplatin only, indicating that curcumin could improve the potency of chemotherapy in modulating the tumor microenvironment and reversing drug resistance. Curcumin is amphipathic and poorly soluble in water; thus, different nanoformulations, including curcumin nanoparticles, are prepared to improve its solubility and targeting abilities. In a study, curcumin nanoparticles provided a better therapeutic effect than curcumin‐free when tested on a mouse model with pancreatic cancer. The nanoparticle formulation had better tumor uptake and circulation time and, therefore, better tumor growth suppression and increased survival rate of the mice (Kunnumakkara et al. 2017). Liposomes and micelles, like other nano‐carriers, have also been explored for the improvement of the anticancer activity of curcumin. In a study, curcumin‐loaded liposomes were affirmed to have the potential to inhibit melanoma growth in a mouse model. Curcumin‐containing liposomes significantly suppressed tumor growth and exhibited improved bioavailability, distribution, and pharmacokinetics compared with curcumin‐free liposomes, indicating that nanotechnology may be used effectively to increase curcumin's therapeutic value in vivo. The molecular targets and mechanisms of curcumin action in cancer cells have been identified in various in vitro experiments, and its therapeutic potential has also been shown in in vivo models of cancer development (Willenbacher et al. 2019). Curcuminoids showed anticancer activity against different types of cancer as shown in Table 1.

**TABLE 1:** Anticancer mechanism of curcuminoids.

Type of cancer	Component	Dosage	Cell line	Mechanism of action	Citations
Kidney cancer	Curcumin	10–40 μm	Caki‐1 786‐O	Induces Apoptosis Inhibits mTOR	Qiao et al. (2023)
Stomach cancer	Curcumin	10–40 μm	MNK‐45 AGS	Induces Apoptosis Inhibits NF‐κB	Moon (2024)
Prostate cancer	Curcumin	10–25 μm	PC‐3 LNCap	Induces Apoptosis Inhibits androgen receptor signaling	Abd. Wahab et al. (2020)
Liver cancer	Demethyoxycurcumin	10–30 μm	Huh‐7 HepG‐2	Inhibits STAT/JAK pathways	Tomeh et al. (2019)
Breast cancer	Curcumin Demethoxycurcumin	10–50 μm	MDA‐Mb‐231 MCF‐7	Inhibits NF‐ κB Interacts with the ERK/MAPK signaling pathway	Farghadani and Naidu (2021)
Lung cancer	Curcumin	15–30 μm	H1299 A549	Suppresses PI3K/Akt pathways Induces autophagy	Zhang et al. (2015)
Pancreatic cancer	Curcumin	20–50 μm	PANC‐1 MiaPaCa‐2	Inhibits NF‐ κB and STAT3 pathways Blocks Notch1 and TGF‐β signaling pathways	Mansouri et al. (2020)
Brain cancer	Curcumin Demethoxycurcumin	10–50 μm	U251 U87	Induces Apoptosis Suppresses PI3K/Akt pathways	Wong et al. (2021)
Colorectal cancer	Curcumin	20–50 μm	SW480 HCT116	Inhibits Wnt/β‐catenin pathway Induces cell cycle arrest	Kunnumakkara et al. (2017)
Bone cancer	Bisdemethoxycurcumin	10–40 μm	Soas‐2 MG‐63	Induces Apoptosis Inhibits NF‐κB	Huang et al. (2020)
Thyroid cancer	Curcumin	10–50 μm	TPC‐1 BCPAP	Induces Apoptosis Inhibits NF‐κB	Perna et al. (2018)
Blood cancer	Curcumin Demethoxycurcumin	5–20 μm	HL‐60 K562 U937	Suppresses PI3K/Akt pathways Induces Apoptosis Inhibits NF‐κB	Joshi et al. (2021)
Uterine cancer	Curcumin	10–30 μm	HEC‐A1 Ishikwa	Suppresses PI3K/Akt pathways Induces Apoptosis	Jahanbakhshi et al. (2021)
Cervical cancer	Curcumin	10–30 μm	SiHa HeLa	Induces Apoptosis Inhibits NF‐κB	He et al. (2019)

## 5 Brain Cancer

Of all diseases, brain cancer and particularly Glioblastoma pose many challenges due to its invasiveness and inherent resistance to conventional treatment. In recent scientific findings, curcuminoids have been reported to possess direct anti‐neoplastic properties against brain cancer, including immune regulation, apoptosis, metastasis inhibition, and cell cycle inhibition. Due to different molecular targets involving signaling pathways, curcuminoids are shown to control the growth of brain cancer cells. One of the principles is the inhibition of the PI3K/Akt/mTOR pathways, critical in cell growth and survival of glioblastoma, and are hyper‐expressed in the majority of cases. Many signaling pathways are dysregulated in glioblastoma, of which the most critical is the PI3K/Akt/mTOR signaling that controls cell proliferation and survival. Scientists have shown that curcumin can suppress Akt phosphorylation effectively, thereby lessening the phosphorylation of some downstream targets such as mTOR and CDKs for the progression of the cell cycle (Zhang, Polyakov, et al. 2018; Zhang, Zhang, et al. 2018). The current study also proves that curcumin selectively induced cell cycle arrest of glioma cells in the G2/M phase, which is due to the reduction in the expression of CDK1 and cyclin B1 (Banik et al. 2020).

The same molecular targets have been identified to be regulated by both DMC and BDMC with antiproliferative activity. New investigations have shown that, although they differ in terms of physicochemical properties by the number of methoxy groups, curcumin analogs have the potential to inhibit glioblastoma cells' proliferation similarly to curcumin (Mishra et al. 2019). Curcuminoids more specifically induce both intracellular via the mitochondria and extracellular via death receptor signaling pathways to apoptosis in the brain cancer cells. Through the increased Bax/Bcl‐2 apoptotic ratio, curcumin activates the intrinsic pathway. This results in cytochrome c release, mitochondrial dysfunction, and activation of caspase‐9 activity. Curcumin has also been described to enhance the extrinsic route through an increase in the death receptors, for example, Fas, which activates caspase 8 and the executioner pro‐caspases (Li et al. 2021). The glioblastoma cells are equally capable of depleting their own source of nutrition to die via apoptosis when instructed to do so by BDMC, which, when tested, was also seen to induce the growth of the p53 gene. Curcuminoids are confirmed to play an essential role in the regulation of apoptotic genes in glioblastoma, where they function as potent inducers of programmed cell death (Singh et al. 2022).

The major complication of cerebral malignancy treatment is its invasiveness. As the disintegration of the extracellular matrix (ECM) and tumor invasion require matrix metalloproteinases (MMPs), mainly MMP‐2 and MMP‐9, curcumin has demonstrated the ability to inhibit these processes. Downregulation of cell migration and invasion in glioblastoma has been linked to curcumin inhibition of MMP activity (Li et al. 2022). It also affects epithelial‐mesenchymal transition, which has been used in the context of metastatic cancer. Curcumin causes suppression of invasion of glioblastoma cells by increasing epithelial markers like E‐cadherin and decreasing mesenchymal markers such as vimentin and N‐cadherin. Furthermore, curcuminoid changes the tumor microenvironment, which is essential for the progression of glioblastoma through the reduction of inflammation (Sung et al. 2021). The result of many studies indicated that curcumin inhibits NF‐κB‐mediated cytokines such as TNF‐α and IL‐6 synthesis (Wang et al. 2024). Thus, curcumin decreases inflammation responsible for the growth and survival of cancer cells by inhibiting NF‐κB. In the latest research, curcumin has been reported to possess immunomodulatory properties in brain cancer cells. These effects include enhancement and production of natural killer (NK) cells and cytotoxic T lymphocytes, which are key components in assembling the immune barrier against cancer cells (He et al. 2022). This anti‐rheumatic property of curcumin is also of great value in the control of brain cancer, particularly on account of its ability to modulate immunologic reactions. In particular, glioblastoma leads to over‐activation of the PI3K/Akt/mTOR signaling pathway, which in turn leads to uncontrolled cell survival, growth, and proliferation. It has been shown that curcuminoids inhibit Akt via the reduction of Akt phosphorylation, and along with it, the mTOR signaling. This inhibition leads to apoptosis and stops glioblastoma cells from multiplying. For instance, curcumin has been described to reduce cyclin B1 and CDK1, which directly affect tumor growth and exhibit G2/M cell cycle arrest (Banik et al. 2020).

The Wnt/β‐catenin signaling is recognized as one of the critical routes required to preserve stem cells, which is manifested in the enhancement of cancer cell invasive and proliferative ability due to its aberrant activation in cancer, including brain cancer. Curcuminoids down‐regulate the nuclear β‐catenin to inhibit the active Wnt/β‐catenin signaling. This inhibition controls the development of tumors and prevents the expansion of glioblastoma stem cells. Researchers have observed that curcumin reduces the β‐catenin level, which in turn reduces the invasiveness of glioblastoma cells (Jiang et al. 2019). Scientists investigated that curcumin usage involved a 54‐year‐old male patient with persistent glioblastoma who was a surgical, radiation, and chemotherapy survivor. This patient was a participant in a clinical trial in which a dose of 8 g of curcumin daily was provided. While undergoing curcumin supplementation for half a year, the patient's MRI demonstrated a significant decrease in the size of the tumor, and the patient complained less about neurological signs and felt better overall. Consequently, this example showed the potential of curcumin as a glioblastoma adjuvant treatment (Zhang, Polyakov, et al. 2018; Zhang, Zhang, et al. 2018). In another study, the researchers investigated the effect of demethoxycurcumin in a nine‐year‐old patient who had been admitted with glioblastoma. When the patient had chemotherapy and the tumor was taken out, the dosage of 5 g of DMC was administered daily. Six months later, scans showed no visible progress and a stable condition. The patient also reported fewer treatment‐related adverse effects. The study indicated that DMC may be safer for curcumin in patients with pediatric glioblastoma because of the compound's strong anticancer effects and minimal side effects (Singh et al. 2022).

The researchers looked into temozolomide treatment and curcumin treatment on a 47‐year‐old female GBM patient. The patient received regular chemotherapy, and 6 g/day of curcumin was administered to her. Scans performed after an interval of 4 months of the patient's treatment showed the tumor had reduced significantly, and the patient experienced fewer side effects compared to chemotherapy alone. This study suggested that the addition of curcumin could enhance temozolomide's treatment efficacy in glioblastoma due to its potential for reducing inflammation and promoting apoptosis. Curcuminoids can't be completely incorporated into clinical practice, though, until issues like low bioavailability are resolved. These restrictions might be removed and the entire therapeutic potential of curcuminoids in brain tumors revealed by developments in drug delivery technologies, such as nanoparticle formulations (Afshari et al. 2021).

## 6 Prostate Cancer

Prostate cancer is the second most frequent type of cancer among men worldwide. As reported by the “Global Cancer Observatory (GLOBOCAN)” study, there were over 1.4 million new cases of prostate cancer in 2020, which made up 14.1% of men's cancer cases around the world (Rawla 2019). Males over 65 have the highest rates of incidence for this cancer, which tends to affect older males. The highest rates of prostate cancer diagnosis are found in industrialized areas, especially in North America and certain parts of Europe. On the other hand, Asia and sub‐Saharan Africa appear to have reduced rates of incidence (Siegel et al. 2021). The mortality rates from prostate cancer continue to be problematic, particularly in regions where access to screening and treatment options is limited, despite regional differences in incidence (Ferlay et al. 2020). Although prostate‐specific antigen (PSA) testing has significantly helped in the early identification of the disease in some countries, the uncertainties of overdiagnosis and overtreatment are still concerning (Mottet et al. 2020). Individuals who have a family history of prostate cancer are twice as likely to develop prostate cancer later. Moreover, African‐American men experience both higher incidence and mortality from prostate cancer at a greater rate than men from other backgrounds (Carter et al. 2018). Investigations have thoroughly examined the bioactive components called curcuminoids for their ability to treat cancer. Curcumin is the foremost investigated of all of them and has been proven to modify signaling pathways associated with the growth and evolution of prostate cancer. Curcumin has been shown to have potent anti‐inflammatory, antioxidant, and anti‐proliferative properties. The study targets specifically pathways including NF‐κB, Akt/mTOR, and androgen receptor (AR) signaling, which are necessary for the initiation and progression of prostate cancer (Panahi et al. 2018).

The transcription factor, the Nuclear Factor‐kappa B (NF‐κB), is responsible for both immunological responses and inflammation and is essential for cell viability. The abnormal activation of NF‐κB has been related to metastasis development, tumor formation, and resistance against apoptosis in prostate cancer (Kumar et al. 2021). Inhibition of phosphorylation and degradation of IκB by curcumin prevents the nuclear translocation and activation by NF‐κB of genes that are both pro‐inflammatory and anti‐apoptotic (Gupta et al. 2019). Inhibition causes a fall in prostate cancer cells of proteins critical for cell survival, such as Bcl‐2 and Bcl‐xL, which ultimately encourages apoptosis. Controlling cell growth, survival, and metabolism is the role of the Akt/mTOR signal system. There is frequent hyper‐activation in prostate cancer, which supports tumor growth and higher treatment resistance (Cui et al. 2024). Evidence demonstrated that curcumin stops Akt phosphorylation, leading to diminished mTOR downstream signaling (Prasad et al. 2019). Those prostate cancer cells that have this mechanism turned off have demonstrated a rise in autophagy along with less tumor progression. Also, pre‐clinical findings showed that curcumin may enhance the effect of chemotherapy on prostate cancer cells by interfering with the Akt/mTOR signaling pathway. As a result, curcumin may act as an adjuvant therapy to improve the efficiency of traditional treatments (Bagheri et al. 2022). Androgen receptor (AR) signaling plays a significant role in the proliferation of prostate cancer, especially when it is in early stages and sensitive to hormones. The encoding of genes that stimulate prostate cell growth begins when androgens like testosterone bind to ARs. Research revealed that curcumin decreases PSA (prostate‐specific antigen) values and suppresses AR expression, which are both important markers of the progression of prostate cancer. Curcumin obstructs AR signaling and thus decreases the proliferation of prostate cancer cells, while also causing apoptosis and preventing the cell cycle from continuing (Abd. Wahab et al. 2020).

In 2019, clinical research investigated curcumin supplementation in patients with prostate cancer on a radiation therapy regimen. One hundred eighty participants engaged in the study and were split into two groups: a placebo group and a group receiving 3 g of curcumin each day. Patients receiving curcumin had markedly lower PSA levels than the control group members after 3 months. In addition to having lower PSA levels, those in the curcumin group suffered from fewer side effects related to radiation, like inflammation and urological complications. This investigation exhibited the promise of curcumin as a supportive therapy for regulating PSA levels and improving patient outcomes throughout radiation treatment (Wang et al. 2024). A patient aged 65 diagnosed with advanced prostate cancer received curcumin as part of his adjuvant therapy, according to research. The patient's condition exhibited barely any response after a year or more of androgen deprivation therapy (ADT). Their PSA levels decreased remarkably from 20 ng/mL to 8 ng/mL over 6 months after the patient began to take 2 g of curcumin supplementation daily. The patient's quality of life has improved along with a shrinking size of the tumor, as reported by imaging studies. Perhaps the reason that the combination of curcumin and ADT was more successful than ADT by itself is curcumin's ability to change inflammatory pathways and AR signaling (Zoi et al. 2021). A case of a 72‐year‐old prostate cancer patient with metastatic disease was treated with curcumin as described by researchers. The patient presented with extensive metastases and had received prior treatment with several systemic chemotherapeutic regimens. After 6 months of the patient's treatment with the addition of 2 g of curcumin, a 35% decrease in the bone metastases was seen on imaging. Moreover, the patient complained of reduced bone pain and increased physical activity as some of the positive changes that contributed to the quality of life. The global burden of prostate cancer is still high, particularly in elderly patients. As a molecule that can selectively modulate the signaling pathways known to play a role in the progression of prostate cancer, curcumin offers a potential therapy. As such, it has the potential to improve patient outcomes as evidenced by its ability to reduce PSA levels, control tumor progression, and complement radiation and chemotherapy. Curcumin has the potential to be the key to treating prostate cancer; however, more studies need to be conducted to determine how effective it is, especially for those who have advanced or hard‐to‐treat forms of the disease (Schmidt and Figg 2016).

## 7 Breast Cancer

Breast cancer is still the most common cancer type in females across the globe. According to the WHO, about 2.3 million new cases of breast cancer were detected in the global region in 2020, or more than 11.7% of all cancer cases (Arnold et al. 2020). In North America and Western Europe, the prevalence of breast cancer is slightly higher than in other parts of the world. However, due to the shift in lifestyles, diets, and reproductive practices in addition to increased life span, the disease is on the rise among the LMICs (Sung et al. 2021). It is well understood that breast cancer is a genetic and clinical pathologic heterogeneous disease with multiple histopathological and molecular features that determine the treatment response and clinical behavior of the disease (Baliu‐Piqué et al. 2020). Concerning these subtypes, the main factors are defined as the differential expression of the growth factor coupled with hormonal receptors (HR), which are proteins found inside and on the surface of specific cells that bind to hormones and trigger a response, by the presence of progesterone and estrogen receptors (PR and ER) or the amplification/overexpression of the HER2 oncogene (Dai et al. 2017). About two‐thirds of breast cancer is hormone receptor‐positive, meaning the cancer cells have receptors for the hormones estrogen and/or progesterone, and are endocrine sensitive, meaning they can be treated with drugs that block these hormones or their effects, such as aromatase inhibitors and selective ER modulators (Lim et al. 2016). However, in 15%–30% of BC patients, there is an overexpression of HER2, a tyrosine kinase receptor responsible for such actions as cell proliferation and survival. The present data also indicate that the prevalence of HER2‐positivity is higher in HR‐HR‐HR‐malignancies, which is connected with an aggressive condition and an unfavorable prognosis (Schedin et al. 2018).

This is an advantage because now HER2‐targeted therapies bring about much better survival rates in hormone‐independent HER2+ + patients; however, drug‐related side effects are still a challenge. In addition, a more aggressive variant called triple‐negative breast cancer (TNBC) makes up 15%–20% of breast cancer cases overall. TNBC is defined as HER2‐negative and clinically ER‐/PR‐. Compared to other subtypes, TNBC shows a more aggressive clinical course, higher rates of early recurrence, and a high incidence of metastasis to the brain and lungs, and less overall survival. Moreover, the concern is a difficult‐to‐treat population that does not respond to hormone therapy or HER2 receptor‐specific therapy (Bimonte et al. 2020).

Curcuminoids can modify different cancer outcomes due to their role in breast cancer cells through molecular mechanisms. Cancer stem cells are a small group of cells capable of generating tumors, spreading cancer, and causing recurrence. Breast CSCs can be selectively targeted by altering curcuminoids at the Wnt/β‐catenin, Notch, and Hedgehog pathways, all of which are key in maintaining CSC self‐renewal (Li et al. 2018).

Curcuminoids reduce the population of CSCs by blocking these pathways, which in turn enhances the efficacy of conventional treatments such as radiotherapy and chemotherapy (Prasad et al. 2021). Research revealed that curcumin increased the effectiveness of paclitaxel, a standard chemotherapy drug for TNBC. When paclitaxel and curcumin were used concomitantly, the percentage of apoptosis that was observed in the TNBC cells was significantly higher than when paclitaxel was used on its own. In the study, curcumin inhibited the NF‐κB signaling so that more cancer cells took up paclitaxel, leading to apoptosis (Wang, Zhang, et al. 2021; Wang, Cao, et al. 2021; Wang, Liu, et al. 2021). A clinical trial examined the effectiveness of curcumin‐conjugated nanoparticles for handling HER2‐positive breast cancer. HER2‐positive breast cancer entails a situation where the HER2 receptor is overexpressed within the breast tissue, accelerating the division of cells. This study thereby ascertained that curcumin nanoparticles potentiated the inhibition of PI3K/Akt/mTOR signaling stimulated by HER2, reduced cancer cell proliferation, and intensified apoptosis (Sinha et al. 2020). Curcuminoids regulate estrogen‐receptor signaling and prevent HER2‐mediated proliferation. It has been found to have synergistic effects when used together with tamoxifen, which lowered drug resistance (Khan et al. 2023).

A study analyzed the applicability of curcuminoid derivatives, especially demethoxycurcumin, in overcoming the tamoxifen resistance of ER+ + breast cancer patients. DMC was able to suppress the estrogen‐induced proliferation by interacting with the ERK/MAPK signaling pathway and also by reducing the estrogen receptor α (ERα) level (Huang et al. 2023).

## 8 Cervical Cancer

Cervical cancer ranked among the highest incidences of women's diseases in the world. According to the WHO (2020) estimates of 604,127 new cases and 341,831 deaths in 2020, cervical cancer has been ranked fourth among women's cancers by incidence as well as mortality. As curable screening and treatment are inaccessible, it predominantly affects women in low and middle‐income countries. Cervical cancer is mainly associated with HPV; more than 70% of cervical cancer is due to HPV types 16 and 18. Therapies that are relevant to the eradication of HPV oncogenesis are essential, as cervical cancer is prevalent (Crosbie et al. 2019). For instance, in the cervix, this narrow zone is known as the transformation zone, where squamous and glandular epithelium come side by side, and cervical cancer often originates. There are two main histological kinds of cervical cancer: Adenocarcinoma, which accounts for 20%–25%, while squamous cell carcinoma constitutes 70%–80% of the rates of occurrence of the cancer. They are cervical intraepithelial neoplasia (CIN) that progresses to invasive carcinoma if not treated. There are multiple cellular changes that are implicated in the pathophysiology: proliferation, apoptosis, new blood vessel formation, and metastasis (Burd 2019). This paper explores cervical cancer and its development through signaling pathways. These include the Wnt/β‐catenin, NF‐κB, MAPK/ERK, and PI3K/AKT/mTOR signaling pathways. Based on the analysis of HPV onco‐proteins E6 and E7, the proteins exhibit a crucial role in influencing mutual host cell protein–protein interactions such as p53 and retinoblastoma (Rb) required for cellular transformation and oncogenesis (Wilken et al. 2017). Thus, the inhibitory effects of curcuminoids on HPV E6 and E7 oncoproteins expression in cervical cancer cells have been shown. These events lead to the regulation of the cell cycle and cause apoptosis through antagonism of altered functions of tumor‐suppressing molecules like Rb and p53 (Nair et al. 2019). A clinical trial investigated the effects of a vaginal moisturizer with curcumin in women affected by HPV‐positive cervical intraepithelial neoplasia (CIN). Data from this research revealed that 70% of patients had their HPV viral load reduced and observed enhanced histology of CIN lesions after 12 weeks of treatment. The ability of curcuminoids to modulate the host immune response, besides their antiviral and anti‐inflammatory properties, explained their therapeutic benefits (Gattoc et al. 2016). The researchers have proved that curcumin exhibits AP‐1 inhibitory activity and reduces the transcription level of HPV18, which alters the fractionating pattern of fra‐1 and c‐fos in cervical cancer cells. Furthermore, it is also active against the mitochondrial pathway and suppresses COX‐2 and iNOS and reduces ERK and Ras signaling pathways and telomerase activities of cancer cells, particularly the HPV cancer cells (Mishra et al. 2019).

Altogether, the in vivo analysis demonstrated that curcumin and BDMC have strong anti‐angiogenic and anti‐metastatic effects on cervical carcinoma in mice. Cervical tumors were induced by implanting CaSki cells into mice for 5 weeks; the mice were treated with either BDMC or curcumin by intraperitoneal route. This was evidenced by the fact that curcuminoid treatment for cancer patients in the study significantly lowered MVD, which is evidence of angiogenesis suppression. Also, the curcuminoids‐treated group showed significantly (*p* < 0.05) a smaller number of metastatic nodules compared with the control group of animals. The anti‐metastatic effects were remarked to reduce MMP‐9 level and increase TIMP‐1 level, and the anti‐angiogenic effects were attributed to low levels of VEGF and HIF‐1α. The combined effect of using PD‐L1 antibodies and curcumin in a cervical cancer‐bearing mouse model. Mice bearing tumor‐carrying TC‐1 cells, a mouse cervical cancer cell line, were treated with curcumin at 200 mg/kg and anti‐PD‐L1 antibodies for 4 weeks. This combination treatment was clearly more effective than monotherapy or control groups, as the growth of the tumor was markedly slowed down. Curcumin also increased the migration of CD8+ T cells into the tumor site and decreased the level of PD‐L1 in tumor cells, as stated in the study, which gave an indication of improved immune response against tumor cells (Liao et al. 2018).

## 9 Uterine Cancer

The most common gynecological cancer worldwide is uterine cancer, and notably, endometrial cancer is the leading type. According to GLOBOCAN reports of 2020, uterine cancer accounted for more than 417,367 new cases and 97,370 deaths globally. Incidence of the disease is gradually increasing and higher in developed countries due to changed lifestyle regarding obesity and reproductive behavior (Ferlay et al. 2024). This disease is most prevalent in postmenopausal women, with a diagnosis at approximately 60 years old. However, studies show that the proportion of premenopausal women is younger, perhaps indicating an increasing trend of metabolic syndrome and obesity. Non‐metastatic endometrial cancer diagnosed at its preliminary stage tends to exhibit a favorable prognosis. Moreover, those cases that have advanced or relapse do not usually respond well to conventional therapies that require the administration of curcuminoid therapies (Singla et al. 2022). Endometrial carcinomas arise from uterine cancer that tends to begin in the upper layer of the endometrial cells. There are mainly two types: Type I (endometrioid adenocarcinomas) and Type II (non‐endometrioid, serous or clear cell carcinomas). Type II tumors are generally invasive and often associated with chemo resistance, whereas Type I cancers are estrogen and progesterone receptor positive and are usually associated with a good prognosis (Morice et al. 2016). A process called angiogenesis is the formation of new blood vessels and is critical to both tumor growth and cancerous movement to other locations. Studies indicated that curcuminoids reduce the secretion of vascular endothelial growth factor (VEGF), preventing new blood vessel formation and reducing the chances of uterine cancer cells metastasizing (Wang and Yi 2018).

Curcuminoids and their constituents' effects on endometrial cancer tumor cell lines, Ishikawa and KLE, were investigated in vitro. The study revealed that curcumin, demethoxycurcumin, and bisdemethoxycurcumin inhibited cell growth dose dependently and induced apoptosis. Bax and caspase‐3 were upregulated, and Bcl‐2 and survivin were downregulated to give this impression. The study also confirmed that these anticancer effects were linked with the inhibition of the PI3K/AKT/mTOR pathway, which is commonly upregulated in endometrial cancers (Liu et al. 2017). An interventional clinical study with 20 post‐surgery chemotherapeutic women with advanced uterine cancer collected data concerning their QoL after consuming curcumin. In a double‐blinded, placebo‐controlled clinical trial, the patients were assigned randomly to receive 4 g/day curcumin or placebo for 6 months. This research found that in the curcumin group, the patients' physical as well as mental health was comparatively much better than that of the placebo group of patients. Additionally, positive correlations between curcumin administration and reduced fatigue, as well as minimization of gastrointestinal side effects resulting from chemotherapy, were observed. These outcomes suggested that curcumin may aid in the improvement of the quality of life in those with uterine cancer because it successfully lowers some of the adverse effects of chemotherapy (Kumar et al. 2017). A case study reported the effects of curcumin supplementation on a 56‐year‐old postmenopausal lady diagnosed with early‐stage endometrial cancer. Curcumin was administered at a dose of 3 g daily for 6 months as an adjuvant strategy to surgical excision of grade 1 endometrioid cancer. Over this time, the patient demonstrated no signs of tumor reappearance and did not have any adverse effects (Liu et al. 2017).

## 10 Kidney Cancer

Kidney cancer, also known as Renal Cell Carcinoma (RCCs), is one of the ten most prevalent types of cancer that are diagnosed globally. Some 431,270 new cases were detected in 2020 and made up 2.2% of all cancers with regard to incidence (Sung et al. 2021). RCC most commonly affects older persons, with the median age at diagnosis being 64 years. Significantly, kidney cancer exhibits interregional heterogeneity despite a global trend over the past few decades toward a higher incidence of the disease. Africa and Asia record the lowest percentage of detection, while North America and certain regions of Europe record the highest percentage of detection (Capitanio and Montorsi 2016).

Numerous statistical analyses indicated that obesity, smoking, high blood pressure, and other environmental pollutants are among the many factors that contribute to kidney cancer. Genetics also has a larger risk involvement, as demonstrated by conditions like Von Hippel–Lindau (VHL), which predisposes individuals to RCC (Linehan and Ricketts 2019). Despite growing knowledge and research on the etiology, diagnosis, and treatment of kidney cancer, it is depressing to see that the disease still has a high cancer‐related death rate, particularly when it has progressed to an advanced stage. It is essential to discover kidney cancer early enough to improve survival rates, as the five‐year survival rate for metastatic kidney cancer is about 12%. In contrast, the rate for localized kidney cancer is expected to be approximately 93% (American Cancer Society 2022). Many genetic and molecular alterations discovered in kidney cancer cells have factors that help support the growth of the tumor and cancer metastasis. The second primary genetic abnormality linked with kidney cancer is the inactivity of the Von Hippel–Lindau (VHL) tumor suppressor gene. Consequently, hypoxia‐inducible factors (HIFs) are stabilized primarily by HIF‐1α, and HIF‐2α raises vascular endothelial growth factor (VEGF), resulting in angiogenesis. This helps in the development and extension of kidney tumors since it increases their hyper‐vascularity (Linehan and Ricketts 2019). These natural polyphenolic compounds, known as curcuminoids, exhibit anticancer activities against numerous types of cancer, such as kidney cancer. The main curcumin, which is an essential active compound in turmeric, along with other curcumin derivatives, that is, demethoxycurcumin and bisdemethoxycurcumin, influence several molecular targets associated with carcinogenesis, cell death, and metastasis to exhibit anticancer activity (Goel et al. 2022). In ccRCC, the loss of VHL gene function leads to the stabilization of hypoxia‐inducible factors (HIF‐1α/2α/2α) and incites angiogenesis and tumor growth. The researcher has shown that curcuminoids reduce VHL‐HIF signaling by inhibiting the synthesis of HIF‐1α, which in turn ultimately leads to a decrease in VEGF synthesis and angiogenesis. In this regard, curcuminoids were especially significant for their ability to suppress the angiogenic phenotype of kidney cancer (Kumar et al. 2020). PI3K/AKT is a signaling pathway that is important for metabolism, cell proliferation, and cell survival. Kidney cancer, in particular, often experiences constitutive activation of this pathway as a consequence of mutations in the VHL and other tumor suppressor genes. A researcher studied a case of a 60‐year‐old man who was diagnosed with metastatic kidney cancer, and he manifested with liver and lung metastases. The patient took curcumin as adjuvant therapy after undergoing regular chemotherapy sessions. Imaging examinations pointed to a decrease in the size of the tumor, and the general tumor markers of the patient lowered significantly over 6 months. Molecular analysis indicated that the anti‐inflammatory cytokine was decreased, and the activity of the nuclear factor κ‐light‐chain‐enhancer of activated B cells (NF‐κB) pathway was reduced. This example shows curcuminoids enhance the effects of chemotherapy by promoting intake, inducing apoptosis of kidney cancer cells, and reducing inflammation (Goel et al. 2022).

In another study, a female patient with stage II kidney cancer consumed curcumin supplements and altered her diet. After 12 months of curcumin administration, molecular assays indicated reduced PI3K/AKT signaling and increased apoptosis in tumor specimens, as well as no indication of tumor formation on imaging studies after curcumin supplementation. This case illustrated that curcuminoids may inhibit the growth and progression of kidney cancer tumors in their preliminary stage (Liu et al. 2022). A researcher examined the chemopreventive effect of curcumin in a 65‐year‐old man who received chemotherapy/nephrectomy for an asymptomatic right flank recurrence of kidney cancer. He started taking the combination of demethoxycurcumin and bis‐demethoxycurcumin. The size of the tumor was considerably decreased to a size of 4 cm in approximately 9 months, and PET analysis showed low MAPK level and low HIF‐VHL level. In this case, the patient experienced enhanced quality of life and reduced disease burden by the use of curcuminoids, so it may potentially help prevent the recurrence of kidney cancer. Overall, these studies showed that curcuminoids can induce apoptosis, control cell division, and decrease levels of angiogenesis. The case histories emphasize the application of curcuminoids as additional therapy for the patients and give an understanding of the role in the treatment of early kidney cancer and the late‐stage cancer (Kumar et al. 2020).

## 11 Bladder Cancer

Bladder cancer is considered one of the most common urological malignancies worldwide, accounting for 90% of urinary tract tumors and 3% of all cancers. A survey by the American Cancer Society estimated that bladder cancer would cause 17,000 deaths and 81,000 new cases in the United States alone in 2023. Bladder cancer is a frequent urological malignancy in older adults worldwide, and its incidence varies slightly depending on age, sex, and geographic location. In the United States alone, bladder cancer was predicted to be responsible for 17,000 fatalities and 81,000 new cases in 2023, according to the American Cancer Society. Bladder cancer incidence varies by area and demographics; older persons, especially men, have greater rates than females. Known predisposing conditions include genetic factors, chronic inflammation of the bladder, smoking, and contact with particular chemicals (Ploeg et al. 2019). The cells lining the bladder and the layer responsible for the bladder's capability to contain urine contribute most to bladder cancer. In bladder cancer, the following cellular phenotypes were found to be important. Urothelial carcinoma represents the majority of bladder malignancies, and can be subtyped as either transitional cell carcinoma or squamous cell carcinoma. Treating these cancer cells is challenging because they can invade adjacent organs and are characterized by significant heterogeneity most of the time (Kaseb and Aeddula 2020). According to recent research, bladder cancer can be divided into two subtypes: basal and luminal, which are recognized to exhibit divergent biological behaviors and prognosis. Although luminal cancers are known to start in tissues with longer proliferative rates, basal cancers begin in rapidly dividing cells and are often associated with a poor outcome (Dobruch and Oszczudłowski 2021). There is strong evidence of cancer stem cells with the potential to proliferate, disseminate, and relapse in bladder cancer. These cells possess greater potentials for survival as well as replication and are recognized by way of specific area codes such as CD44 as well as CD133 (Abugomaa et al. 2020). Curcuminoids' data show anticancer effects of curcuminoids on PI3K/AKT/mTOR signaling and increased apoptosis and decreased proliferation rates of bladder cancer cells. A change in cyclins and cyclin‐dependent kinases (CDKs) occurrence is affected by curcumin since it hinders the activity of cysts, and reduces the multiplication of bladder cancer cells (Wang 2016). Researchers used an orthotropic mouse model of bladder cancer and investigated the therapeutic efficacy of curcumin. The mice received curcumin at a dose of 100 mg/kg/day for 4 weeks before the administration of bladder cancer cells. Tumor mass was determined and weighed, and the volume of the tumor was also calculated. The tumor weight and volume were also reduced in curcumin‐treated mice compared to the control group. Lack of angiogenesis and reduced cell proliferation were also confirmed by histological analysis in bladder cancer treatment by using candesartan, thus confirming the blockage of cancer progression (Tuli 2022). To experimentally verify the effects of curcumin on the progression of bladder cancer in genetically modified mice vulnerable to cancer. After an initial treatment at the start of the tumor in mice, 200 mg/kg/day curcumin was administered for 6 weeks. Tumor size, rate of onset, and molecular actions were assessed. Curcumin significantly blunted both bladder tumor occurrence and tumor progression. According to the activity studies of molecular markers, there were increased profiles of apoptosis‐related proteins and inhibited NF‐κB (Giordano and Tommonaro 2019).

## 12 Liver Cancer

It is observed that in 2020, more than 830,000 people died from liver cancer all over the world (Bray et al. 2024). In recent years, the increase in the incidence of liver cancer has been observed mainly due to increasing rates of metabolic syndrome and non‐alcoholic fatty liver disease (NAFLD) in Western countries. The liver's principal operational unit is hepatocytes, which form the primary focus of liver cancer. HCC is derived from hepatocytes, and liver cancer accounts for 75%–85% of all cancerous growths in the liver. Hepatoblastoma, a rare cancer that affects the liver area of the body in children, and cholangiocarcinoma, a liver cancer that arises from the borderline cells, bile duct cells, are other examples. A wide variety of molecular alterations take place during the progression of liver cancer, including genetic and epigenetic alterations leading to cell proliferation, angiogenesis, and metastasis (Forner et al. 2019). In liver cancer, the TGF‐β/SMAD pathway acts in two ways: addiction and resistance. In the initial stages of tumor development, it arrests cancerous growth by inhibiting cell replication. It promotes invasion, metastasis, and the conversion of epithelial cells to mesenchymal cells (EMT) at the later stage.

Curcuminoids reduce phosphorylation of SMAD2/3 and TGF‐β levels and reduce EMT and metastasis through regulation of TGF‐β/SMAD (Wang, Zhang, et al. 2021; Wang, Cao, et al. 2021; Wang, Liu, et al. 2021). There is also the need to cross signal to the nucleus and activate gene transcriptions, the Janus kinase (JAK)/signal transducer and activator of transcription (STAT) pathway. In liver cancer, it is revealed that there is an association between liver cancer aggravation, angiogenesis, and immune escape, and JAK/STAT3 hyperactivation. Curcumin and demethoxycurcumin suppressed STAT3 phosphorylation and nuclear translocation and consequently caused decreased gene transcription of those genes involved in cell proliferation and survival (Wang et al. 2019).

A study was conducted to compare the impact of demethoxycurcumin on HCC cells. The researchers found that demethoxycurcumin inhibited the cell growth and survival of the HCC cells by increasing the activation levels of caspase‐3 and decreasing the levels of cyclin D1, leading to implications of cell cycle arrest and apoptosis. The analysis on liver cancer made by the researcher concluded that demethoxycurcumin has potential in the treatment of liver cancer. Research was conducted to investigate the effect of bismethoxycurcumin for its anticancer potential to treat liver cancer. In order to reduce tumor growth and metastasis, bismethoxycurcumin was shown to suppress both the epithelial‐mesenchymal transition and the synthesis of MMP‐9 proteins. Furthermore, the study demonstrated that bismethoxycurcumin inhibited the PI3K/AKT/mTOR pathway's activity. These findings supported the potential use of curcuminoids in combination with conventional therapies as well as stand‐alone treatment for liver cancer. More study and clinical trials are required to confirm these results and investigate their application in the field of medicine (Wang, Zhang, et al. 2021; Wang, Cao, et al. 2021; Wang, Liu, et al. 2021).

## 13 Lung Cancer

Lung cancer remains a leading type of cancer that causes death globally; the global mortality from lung cancer is over 1.8 million. Lung cancer's primary subtypes include small cell lung cancer, characterized by fast metastasis as well as increased malignancy, and non‐small cell lung cancer, which constitutes about 85% of cases. Curcuminoids are defined as the active principal group of curcumin, demethoxycurcumin, and bismethoxycurcumin, and all of them have shown significant effects against lung cancer proliferation through apoptosis, signal transduction pathways, and metastasis regulation. Lung cancer was estimated to have contributed to about 11.4% of new cancer cases and 18% of cancer mortalities across the globe in 2020, fulfilling the second most common cancer in the world and the leading cause of cancer death (Sung et al. 2021). A large number of people die from it because it usually progresses to a late stage, particularly in non‐small cell lung cancer. Genetic predispositions, smoking, and exposure to environmental carcinogens—these are the three primary risk factors of lung cancer. About lung cancer, cancer incidence is increasing in low and middle‐income countries, even though it is decreasing in many high‐income countries due to a reduction in smoking (Bray et al. 2022). Most cases of primary lung cancer affect the epithelium, which lines the airways. Lung cancer positive for the cic (cells in ink) test is globally divided into three groups, which include large cell carcinoma, squamous cell carcinoma, and adenocarcinoma, all of which are categorized under NSCLC as they have similar characteristics. All the types of cancer that begin in the bronchus area can be subdivided. Adenocarcinoma most often originates from glandular cells, whereas squamous cell carcinoma begins from flat cells called the bronchial cells. Neuroendocrine cells are the origin of small‐cell lung cancer, characterized by rapid onset and metastasis. These subtypes receive therapies differently because of the various molecular and genetic characteristics that define them (Husain et al. 2018). There is evidence that shows that curcumin suppresses the EGFR and AKT/mTOR pathways in non‐small‐cell lung cancer (NSCLC). In a 2022 meta‐analysis, it was confirmed that there was significant tumor volume and oxidative biomarker reduction in preclinical NSCLC models (Zhang et al. 2022). Clinical trials are being conducted to determine the safety of using standard chemotherapy.

Previous experimental studies on several types of lung cancer cell lines, such as adeno‐carcinomic (A549), human non‐small cell lung cancer (H1299), and large cell carcinoma (H460), all prove that curcuminoids possess significant anti‐proliferative and pro‐apoptotic effects. Curcuminoids achieve this by increasing the expression of pro‐death proteins such as Bax and cleaved caspase‐3, and decreasing the expression of anti‐death proteins such as Bcl‐2, leading to cell cycle arrest and apoptosis. When demethoxycurcumin was used on A549 cells, this was proven in a study to reduce cell growth in a dose‐dependent manner through G2/M cell cycle arrest. The findings of the current research revealed that demethoxycurcumin elicited its anti‐proliferative effect through modulating the cyclins and CDKs, which are responsible for cell cycle control (Man et al. 2018). In research, H460 cells that have been exposed to bismethoxycurcumin display lower capability of invasion and migration. This was associated with a reversal of EMT as determined by the up‐regulation of the epithelial marker E‐cadherin and a down‐regulation of MMP‐2, MMP‐9, and vimentin, a mesenchymal marker (Khan et al. 2018).

## 14 Pancreatic Cancer

Pancreatic cancer is one of the common cancers; only 5%–9% of patients survive for 5 years after diagnosis. About 2030, scientists consider it to be the oncological death rate is seventh in the world and the second in the United States (Siegel et al. 2021). The threat is also low because often patients have early metastases; these tumors are diagnosed at a late stage and are resistant to traditional therapy. Researches done in the past years are centered on natural compounds like curcuminoids due to their anticancer activity.

Pancreatic cancer is a significant health problem because it accounts for 4.7% of cancer mortalities and approximately 3.2% of all cancers globally (Bray et al. 2022).

While this disease remains undetectable in its early stages and there are no special tests for the detection of the disease, it is often diagnosed at a late stage. About 95% of pancreatic cancers are of the category known as pancreatic ductal adenocarcinoma (PDAC). They include diabetes, obesity, smoking, and chronic pancreatitis, as well as inherited factors. These diagnostic techniques suggested that new therapeutic approaches must be explored since incidence and death rates are increasing in HI and LIM despite the availability of sufficient resources.

PDAC arises due to pancreatic cancer arising from the epithelial cells that form the lining of the pancreatic ducts. The first subtypes derived from acinar and neuroendocrine are acinar cell carcinoma and neuroendocrine cancers. It has become apparent that PDAC is characterized by high desmoplasia and a rich stromal compartment that sustains immune escape and resistance to chemotherapy. The emergent cancer cell involving immune cells, fibroblasts, matrix proteins, and other partners are factors that make cancer more aggressive (Adamska et al. 2019). The KRAS gene, which is one of the frequently mutated oncogenes in pancreatic cancer, was mutated in 87% of PDACs. More common in codon 12 (e.g., G12D, G12V), the mutations result in the continuous activation of downstream pathways PI3K/AKT/mTOR and RAF/MEK/ERK. Ongoing stimulation of the MAPK/ERK will contribute to the constant proliferation of cells and the ability of the cells to attack spontaneously. This route affects cell development, survival, and metabolism. AKT and mTOR work similarly via a dysregulated cell signal cascade, which leads to cell growth and proliferation and enhances chemoresistance in pancreatic cancer (Mishra et al. 2023).

It was also indicated that the Hedgehog pathway is involved in tissue development and in the embryo. Its activation in pancreatic cancer patients is abnormally high, thus the formation of a stroma for the implantation of the tumor and for promoting the metastasis of the carcinoma. Patched (PTCH) and smoothened (SMO) proteins are membrane‐bound, and when Hh ligands such as Shh are present, they can bind to PTCH, which releases inhibition of SMO, and activate GLI transcription factors downstream. Some factors that are associated with such genes include cell survival, growth factors, as well as smaller molecule signaling known to be more expressive when the factor is present. Phase II studies have investigated vismodegib, an SMO inhibitor, for its efficacy to inhibit the Hedgehog pathway and the cancer‐associated supportive TME (Li, Sun, et al. 2020).

Additionally, luteolin and curcumin together showed encouraging anti‐cancer action against cancer. The wound healing experiment demonstrates the synergistic inhibition of cancer cell migration and proliferation by curcumin and luteolin. Moreover, Notch‐1 and TGF‐β were suppressed in vitro, in xenograft mice, according to protein expression analysis. Based on these findings, it appeared that curcumin and luteolin can effectively inhibit the growth and spread of cancer cells by blocking the Notch1 and TGF‐β signaling pathways. Another study assessed curcumin's anti‐cancer efficacy in combination with aprepitant, a medication recognized for its antitumor qualities on a variety of malignancies (Muñoz and Coveñas 2020).

An analysis performed by using test tubes has explored the feasibility of supplementing conventional chemotherapy agents, including gemcitabine and 5‐fluorouracil. Interactions between some components have been proven to have magnified effects on the curative effects and the reduction of drug resistance of chemotherapy drugs. PANC‐1 cell study indicated that curcumin combined with gemcitabine is more effective in inducing cell death and reducing cell proliferation than the treatment with gemcitabine alone. Surprisingly, it was established that the combined therapy suppressed the NF‐κB signaling, which has been proven to be involved in the chemoresistance of pancreatic cancer (Li, Sun, et al. 2020).

## 15 Colon Cancer

Globally, colorectal cancer is the most common type of cancer. It is the second major cause of mortality in cancer‐related patients and ranked third in its prevalence among people. Worldwide, approximately 1.9 million new cases of colorectal cancer were reported in 2020, causing 935,000 deaths, and became an emerging health concern with severe impacts on public health (Sung et al. 2021). The prevalence of colorectal cancer is different in different regions of the world. Mostly, the people of the developed countries like Australia, the U.S., and other countries of Europe are highly affected by CRC. Fewer patients are seen in African and South Asian regions (Arnold et al. 2020). Instead of modern and advanced methods used for the screening of colorectal cancer, like fecal immunochemical testing and colonoscopy, it is the primary cause of death in the USA in cancer‐related patients (Siegel et al. 2020). Due to the adoption of a western lifestyle, like eating high‐calorie food which is highly processed, no physical activity, and an increasing number of obese people, the prevalence of colorectal cancer is also increasing in developing countries (Arnold et al. 2020). Globally, the incidence of colorectal cancer is increasing due to modern lifestyle trends, similar patterns that are seen in developed countries (Siegel et al. 2020).

Curcuminoids show their effect against cancer by acting on several biological processes like apoptosis, regulation of cell cycle, angiogenesis, and inflammation. The nuclear factor‐kappa B (NF‐κB), Wnt/β‐catenin, phosphatidylinositol‐3‐kinase (PI3K)/AKT, and mitogen‐activated protein kinase (MAPK) are some major pathways that are affected in colon cancer. Adenomatous polyposis coli (APC) gene mutations are the common cause of dysregulation in the Wnt/β‐catenin pathway that is seen in colon cancer. Beta‐catenin accumulation in the nucleus. This mutation triggers the stimulation of the production of cancerous cells, which causes tumor growth (Kumar et al. 2020). Curcuminoids inhibit the Wnt/β‐catenin pathway by increasing the degradation of β‐catenin and decreasing its nuclear translocation. This process helps in the reduction of tumor growth and prevention of metastasis (Yadav et al. 2020). In colon cancer, demethoxycurcumin and bisdemethoxycurcumin molecules have shown strong inhibitory effects on PI3K/AKT and NF‐κB signaling by lowering pro‐inflammatory cytokines and downregulating anti‐apoptotic proteins, causing apoptosis and preventing the growth of cancer cells (Liu et al. 2022).

A researcher examined the effects of curcuminoids on a patient who took a standard treatment in combination with curcumin, who had stage IV colon cancer with liver metastases. A range of imaging tests was conducted in the next 6 months, in which the size of the tumor was observed to be reducing (Gupta et al. 2024). Using molecular analysis, the researcher found increased cancer cell death, decreased levels of pro‐inflammatory cytokines, and decreased activation of the NF‐κB pathway. This example shows that curcuminoids can make chemotherapy better for advanced colon cancer.

Another case in which interventions that include curcumin and dietary changes were used by a female client diagnosed with stage II colon cancer. Another colonoscopy 1 year later did not show any evidence suggesting that the tumor was growing. At the molecular level, increased apoptosis was observed, along with reduced Wnt/β‐catenin signaling in tumor samples. The patient's quality of life has been enhanced. It remains clear that curcuminoids can reduce the growth of tumors in early‐stage colon cancer from this example (Yadav et al. 2020).

In this study, the researcher found that a patient who started therapy with curcumin, demethoxycurcumin, and bisdemethoxycurcumin for the recurrence of colon cancer after surgical intervention and chemotherapy. Tumor size also reduced, and the levels of PI3K/AKT signaling became lower after the next 9 months. The reduction in tumor growth and enhanced comprehensive wellbeing of the uncovered patient support the use of curcuminoids in the treatment of recurrence of colon cancer (Kumar et al. 2020).

## 16 Thyroid Cancer

PTC accounts for 80%–85% of thyroid cancer, and regardless of the stage, most patients with a positive diagnosis have a favorable outcome at first presentation. On the other hand, FTC accounts for about 10%–15% of thyroid cancer cases and is associated with a propensity to metastasize. ATC is a sporadic but regrettably scarce thyroid malignancy type, accounting for only 1%–2% of all thyroid cancer cases, and has been associated with poor survival rates and few therapeutic interventions. MTC, which arises from parafollicular c‐cells, constitutes 3%–4% of thyroid cancer (Lloyd et al. 2017). Curcuminoids interact with various thyroid cancer cells and signaling pathways. These compounds selectively act on PTC, FTC, MTC, and ATC cells, which are malignant thyroid cells, through interference with different biological activities. MTC is often associated with RET proto‐oncogene activating mutations that activate RET/PTC and downstream signal mediators, PI3K/MAPK. These changes enhance tumor growth and metastasis, promote cell proliferation, and modulate apoptosis or cell death settings (Romei and Elisei 2021). Besides that, chromosomal instability is detected in most of the ATC cases; additional genetic anomalies involving TERT, BRAF, and TP53 promoters are present as well. Scientists identified that in ATC cells, differentiation markers are lost and pathways such as NF‐κB are upregulated to gain capabilities for their uncontrollable proliferation coupled with resistance to apoptosis (Zhang et al. 2022). To compare the impact of thymoquinone, curcumin, and vehicle control on thyroid cancer using a mouse model, having transplanted thyroid cancer cells to mice, they treated the animals for 3 weeks with thymoquinone at a dose of 10 mg/kg, curcumin at a dose of 50 mg/kg, or alone. Assessments were made according to tumor burden and histology changes. In contrast to monotherapy, the use of combined therapy significantly decreased the tumor size. The histopathological analysis, for example, revealed a better apoptotic profile and reduced Ki‐67 cell proliferation, indicating that the two compounds had a synergistic effect in discouraging the formation of tumors (Amin et al. 2023). This work studied how curcumin influences the activity of follicular thyroid cancer cells (FTC‐133) in the cell cycle. To FTC‐133 cells, curcumin treatment was performed at a concentration of 0–25 μM for a period of 24 h. Protein expression was analyzed using a Western blot, and cell cycle profiling was done using flow cytometry. Culturing with curcumin led to the suppression of G2/M cell cycle progression as well as downregulation of CDK1 and cyclin B1 proteins. Based on the findings, curcumin inhibited the proliferation of the FTC cell through cell cycle arrest (Guo et al. 2021).

## 17 Skin Cancer

Skin cancer is one of the most common cancers globally and has been more commonly reported in recent decades. Skin cancer is primarily developed from various functions of several cell types present in the skin's epidermal layer. It is usually classified into two primary classes, which are NMSC and melanoma. All kinds of skin cancer are unique in terms of the cells from which they originate and the manner in which they grow, spread, and alter molecularly. They include melanoma, basal cell carcinoma (BCC), and squamous cell carcinoma (SCC). The development of skin cancer all over the world shall be complemented with appreciation and actually contributes to the increase in numbers, such as increased exposure to ultraviolet (UV) and ozone depletion, and an aging population. Current data also show that many skin cancers are not melanoma; actually, BCC and SCC are the most common types of skin cancer. Melanoma, although rare, leads to the majority of skin cancer‐related fatalities (Chen et al. 2020). Melanocytes give rise to the most dangerous type of skin cancer known as melanoma. In some cases, when it is not diagnosed early or when not treated, it tends to localize to other organs rapidly. Defects of the BRAF onco‐protein and NRAS protein that are involved in the MAPK/ERK signaling cascade in tumorigenesis are associated with melanoma. Melanoma progression has also been due to loss of tumor suppressors, for example, PTEN, and changes in the PI3K/AKT pathway (Hwang et al. 2020). A kind of skin cancer called basal cell carcinoma or BCC emerges on areas of the face and neck that have been a target of sun rays. It can rarely spread and takes so much time to grow. Defects in genes such as SMO and PTCH1 that comprise the Hedgehog (HH) pathway are reported to be linked with BCC. It is because of these mutations that tumor development and uncontrolled cell division occur (Xie et al. 2022).

Essentially, the blood vessel lining, called the endothelial layer, comprises endothelial cells and is also a part of the angiogenesis process. Tumor‐associated endothelial cells formed new capillaries that supply tumor nutrients and oxygen and support its growth (Czyz 2019). As a result, a high proportion of melanomas have the mutated BRAF protein, making curcuminoids capable of inhibiting the protein and MAPK/ERK signaling underlying cancer cell proliferation. Kim et al. (2020) indicated that 45 and 60 μM curcumin reduced the viability of SCC‐9 squamous carcinoma cells since it caused apoptosis and cell cycle arrest at the G2/M phase (Kim et al. 2020). To study the impact that bisdemethoxycurcumin has on the human melanoma cells, WM266‐4, with relevance to the cell cycle regulation, cells of WM266‐4 were treated with bisdemethoxycurcumin (0–25 μM) for a whole day. Analysis of the distribution of the cell cycle was conducted using flow cytometry, while the expression of the proteins that regulate the cell cycle was analyzed by Western blot. However, in the WM266‐4 cells, bisdemethoxycurcumin induced cell cycle arrest in the G1 phase of the cell cycle. Treatment promoted the up‐regulation of p21 and p27 but down‐regulation of CDK4 and cyclin D1. This control of cell cycle proteins led to a suppression of cell growth. The present study, therefore, showed that bisdemethoxycurcumin possesses anticancer activity by inducing cell cycle arrest in melanoma cells and can be used as a lead compound for the development of drugs targeting the cell cycle abnormalities in melanoma (Choudhury et al. 2024b, 2024a).

## 18 Bone Cancer

Despite being very uncommon, bone cancer is a dangerous cancer that primarily affects the bones and can have a substantial negative impact on morbidity and death. Osteosarcoma, chondrosarcoma, and Ewing's sarcoma are the three main types of bone cancer that primarily affect the body's long bones, including the femur, tibia, and humerus. Despite making up less than 1% of all cancer cases, bone cancer is actively being researched for new treatment approaches due to its aggressive nature and propensity for metastasis (Geller and Gorlick 2018). Bone malignancies are relatively rare types of cancer, with an incidence rate of 0.9 per 100,000 people every year (WHO 2022). One such kind is osteosarcoma, which usually affects young adults and teenagers. Chondrosarcoma is more prevalent in patients over 40 years of age. Ewing's sarcoma is mainly diagnosed in children and young adults. The prognosis is considerably poorer in metastatic bone malignancies than it is in localized osteosarcoma; about 60%–80% of patients survive to 5 years. Mortality goes in parity with the type, phase, and treatment outcomes (types) (Davis et al. 2019). The white blood corpuscles most affected by bone cancer are osteoblasts that form bones and chondrocytes that help in cartilage formation. Osteosarcoma is derived from osteoblasts, resulting in extremely excessive rates of bone formation due to the resulting malignant cells. Ewing's sarcoma originates from primitive neuroectodermal cells, and chondrosarcoma originates from modified chondrocytes. It grows these cancerous cells, which actually change the typical structure of bones and produce weak and painful bones. Extensibility and invasion to adjacent tissue are expressions of osteosarcoma and chondrosarcoma that are regulated by the Wnt pathway (Martin et al. 2018). A study was conducted to provide evidence that curcuminoids decrease inflammation and inhibit NF‐κB signaling to alter the tumor microenvironment and inhibit further progression of bone cancer (Prasad et al. 2021).

In one clinical trial, curcuminoids were used in the form of a derivative of curcumin on patients with recurrent Ewing's sarcoma. Of the total patients, 30% experienced a decrease in the activation of PI3K/AKT signaling, showing minimal response at 3 months. Curcumin may help as an adjunct therapy for osteosarcoma, as evidenced by a clinical study of the juvenile patient. Serum markers, including alkaline phosphatase and lactate dehydrogenase, involved in bone and tumor metabolism, were reduced with the patient's supplemental curcumin dosage at 1.5 g per day apart from standard treatment (Wang, Zhang, et al. 2021; Wang, Cao, et al. 2021; Wang, Liu, et al. 2021). A 45‐year‐old female breast cancer patient with bone metastases was administered a novel curcumin nano‐encapsulated compound. It indicated that there were fewer metastatic lesions, and it lowered the MMP and increased the bioavailability of the drug derived from this formulation (Sudhesh Dev et al. 2021).

## 19 Blood Cancer

Blood cancer is the uncontrolled increase and development of blood‐forming or immune system cells, which comprise leukemia, lymphoma, and myeloma. Considering their multiple effects on the cell, natural chemicals such as curcuminoids have recently been considered for cancer treatment. Curcumin, demethoxycurcumin (DMC), and bisdemethoxycurcumin (BDMC), which are all curcuminoids used, have enormous potential in activating apoptosis, signaling pathways, and suppression of cancer cells' proliferation. There are over 1 million new cases of blood malignancies diagnosed annually, which puts them as one of the most common forms of cancer. Hodgkin and non‐Hodgkin lymphoma are two types of blood cancer that do not have the same incidence rate. About 8% of the new cases of cancer are leukemia, and about four‐point‐3%, all types of cancers are lymphomas, especially non‐Hodgkin lymphoma. Also, the incidence of multiple myeloma has risen and comprises 1.8% of all cancer cases globally, as reflected by Globocan 2020 (Islami et al. 2022).

Depending on the particular subtype, various types of blood‐forming cells participate in blood malignancies. For instance, the leukocytes, the white part of blood cells, or WBC, can produce more cells in the wrong manner in leukemia. These embryonic cells include myeloblasts in acute myeloid leukemia (AML) and lymphoblasts in acute lymphoblastic leukemia (ALL) (Alaswad et al. 2021). Lymphomas affect B cells and T cells, excluding atypical T cells or plasma cells; however, B cells are particularly targeted by Hodgkin lymphoma. Multiple myeloma is a type of cancer of the plasma cells that secretes an abnormal number of monoclonal proteins and unbalances the normal production of red blood cells. The role of curcuminoids on persistent cancer cells' survival, apoptosis, and proliferation has been investigated profoundly. In turn, curcumin and its main derivatives, such as demethoxycurcumin, actively act on multiple signaling pathways and have pronounced anticancer activity (Kumar and Rajkumar 2018). As shown for demethoxycurcumin, compounds derived from curcumin exert cytotoxic effects against myeloma cells, as well as the inhibition of key signaling pathways, including PI3K/Akt/mTOR and STAT3 in multiple myeloma (Lee et al. 2022). Curcuminoids activate the internal and externally enacted pathways that cause death in leukemia cells. They lead to mitochondrial dysfunctions along with caspase activation and an imbalance in the Bcl‐xs/Bax/Bcl‐2 proteins (Sarkar et al. 2020).

Curcumin can downregulate survival proteins and change NF‐κB signaling in lymphomas, leading to apoptosis (Sevastre et al. 2022). Researchers found that combining curcumin with conventional therapy enhanced its toxicity toward chronic myeloid leukemia cells. In another study, it was found that demethoxycurcumin inhibits oxidative stress and apoptosis and improves the effectiveness of bortezomib in multiple myeloma. These findings show the efficacy of curcuminoids to complement blood malignancy therapies to increase patient survival rates and minimize industrial medicine adverse reactions (Tan and Norhaizan 2019).

## 20 Future Prospect

The prospects of medical research have shown that curcuminoids in general and curcumin in particular could be used in the treatment of cancer and for general well‐being. Due to the variety of ways that curcumin acts on the body, primarily through the regulation of signaling pathways that are critical for cell survival and inflammation, and their antioxidant function, curcuminoids can be considered possible regulators of carcinogenesis and approaches to combating cancer. First of all, further efforts should be made to enhance the bioavailability of curcuminoids. Native curcumin has low solubility, a high rate of metabolism, and a low retention in the body. These characteristics result in sub‐therapeutic plasma levels and fluctuation of pharmacokinetics. Nanoparticles, liposomal, and phospholipid formulations have better penetration and stability, but cross‐studies are challenging due to the difference in formulations in terms of excipients, particle size, and the frequency of administration (Patel et al. 2024). The second problem derived from the technical aspect is related to the increase in curcuminoids' bioavailability. Research carried out in the recent past has indicated that new curcumin formulations such as liposomes, nanoparticles, and micelles can enhance the solubility and stability of curcumin, thereby enhancing its medicinal use. Present‐day clinical trials concerning this modern technique of distribution will provide significant new data regarding their risk–benefit ratios. Examining how several kinds of conventional chemotherapies work together could provide direction on how to synthesize similar combination pharmaceuticals that are at their most effective and have their minimal adverse reactions (Lozano‐Herrera and Luna‐Barcenas 2022). The following Table 2 summarizes representative human trials evaluating curcuminoids and their analogs to promote translational focus.

**TABLE 2:** Clinical evidence supporting the translational potential of curcuminoids.

Cancer type	Formulation/Dose	Sample size	Key outcome measures	Principal findings	References
Prostate cancer	Curcumin capsule (500 mg BID)	40	PSA levels	↓ PSA and AR activity	Ahmed et al. (2021)
Lung cancer	Curcumin + Chemotherapy	52	Toxicity profile, tumor response	Reduced cisplatin toxicity without efficacy loss	Liang et al. (2024)
Breast cancer	Nano‐curcumin (80 mg daily)	70	Cytokines, tumor marker levels	↓ IL‐6 and VEGF	Rahmani et al. (2023)
Pancreatic cancer	Liposomal curcumin (300 mg/m^2^ IV)	36	Tumor response, OS	Stable disease in 20% patients	Bhattacharya et al. (2020)
Colorectal cancer	Curcumin + piperine (2 g + 20 mg daily)	126	Inflammatory markers, CT imaging	↓ CRP, improved QoL scores	Cheng et al. (2022)

Despite notable advances in curcumin delivery systems, the clinical translation of these nanoformulations remains inconsistent. Randomized controlled trials (RCTs) have shown mixed results regarding both effectiveness and safety. For example, Rahmani et al. (2023) conducted a double‐blind RCT on breast cancer patients, showing that nano‐curcumin supplementation significantly lowered inflammatory cytokines and VEGF levels without notable toxicity. Similarly, Bhattacharya et al. (2020) found that liposomal curcumin in advanced pancreatic cancer increased plasma concentrations and was well‐tolerated at therapeutic doses. However, several meta‐analyses and pharmacokinetic studies reveal discrepancies due to differences in formulation design, dosage, and endpoints. While nano‐based systems improve curcumin's systemic exposure, a significant challenge remains in demonstrating consistent clinical benefits across different patient groups. Future research should focus on direct comparison studies of various formulations to establish standardized efficacy and safety profiles for clinical use (Sohn et al. 2021).

However, identifying which cancer types are most responsive to this substance will require a deeper investigation into the molecular pathways influenced by curcuminoids. It is still unclear how curcumin modulates many signaling pathways from NF‐kB to MAPK. This research will make the task of developing targeted therapy simpler as well as help to understand the mechanism of action of curcumin as an anticancer agent. However, finding out which types of cancer are most likely to demonstrate good outcomes to this substance will demand a closer look into what molecular routes are affected by curcuminoids. It is still unclear how curcumin modulates many signaling pathways from NF‐kB to MAPK. This research will make the task of developing targeted therapy simpler as well as help to understand the mechanism of action of curcumin as an anticancer agent. Curcuminoids are a versatile class of phytochemicals with promising but variable therapeutic potential across different cancers. Their effectiveness depends on molecular context, delivery method, and disease subtype, indicating that tailored, cancer‐specific strategies will be more clinically relevant. In conclusion, curcuminoids represent the whole new generation of molecules with unlimited medical applications. As their action plans are revealed with time, and their bioavailability is improved, curcuminoids may turn into invaluable tools in the fight against cancer and other chronic diseases to become the keys to opening the gates to innovative therapeutic approaches using natural sources in the modern environment (Santos et al. 2024).

Although curcuminoids are generally considered safe and well‐tolerated, emerging clinical data reveal specific adverse effects, especially with long‐term or high‐dose use. Reported side effects in clinical studies include gastrointestinal issues such as nausea, diarrhea, and abdominal discomfort, along with temporary increases in liver enzyme levels. In combination therapy, curcumin has occasionally shown mild hepatotoxicity and interacted with chemotherapeutic agents by affecting cytochrome P450 enzymes (Goswami et al. 2022; Jamil et al. 2023). Additionally, long‐term supplementation can impact iron metabolism and lower serum ferritin levels (Hewlings and Kalman 2017). The studies report good tolerability; some nanoparticle‐based formulations caused reversible increases in bilirubin and creatinine in sensitive individuals. Therefore, while curcuminoids have promising therapeutic potential, careful dose adjustment and monitoring are crucial to minimize toxicity, especially in cancer patients on combination regimens (Sahebkar et al. 2021).

## 21 Conclusion

Curcuminoids have remained one of the most widely studied dietary polyphenols as cancer prevention and therapy agents. They provide a wide mechanistic explanation of their pleiotropic regulation of signaling pathways, but the clinical data remain immature. Recent developments in nano‐delivery and combination regimens have started to overcome pharmacokinetic limitations; however, large‐scale, standardized clinical trials are needed to prove efficacy. This new review offers an evidence‐based basis on upcoming translational research and puts curcuminoids into a broader phytochemical framework comprising ellagic acid and naringenin. Their complete therapeutic potential will be achieved through further interdisciplinary cooperation of pharmacologists, oncologists, and formulation scientists.

## Author Contributions

**Ushna Momal:** conceptualization (equal), writing – original draft (equal). **Muhammad Shahbaz:** conceptualization (equal), writing – original draft (equal). **Asfa Perween:** writing – review and editing (equal). **Muhammad Hammad ul Hassan:** data curation (equal), investigation (equal). **Hammad Naeem:** visualization (equal), writing – review and editing (equal). **Zubda Shahid:** data curation (equal), investigation (equal), software (equal). **Muzzamal Hussain:** resources (equal), writing – original draft (equal). **Muhammad Imran:** data curation (equal), methodology (equal). **Suliman A. Alsagaby:** investigation (equal), validation (equal), visualization (equal). **Waleed Al Abdulmonem:** data curation (equal), investigation (equal). **Mohamed A. Abdelgawad:** writing – review and editing (equal). **Mohammed M. Ghoneim:** data curation (equal), investigation (equal), validation (equal), visualization (equal). **Tadesse FentaYehuala:** data curation (equal), investigation (equal), supervision (equal), writing – review and editing (equal). **Samy Selim:** data curation (equal), investigation (equal). **Ehab M. Mostafa:** resources (equal), writing – original draft (equal).

## Funding

The authors have nothing to report.

## Conflicts of Interest

The authors declare no conflicts of interest.

## Data Availability

The data that support the findings of this study are available on request from the corresponding author.
